# Transcriptome sequencing reveals a lncRNA–mRNA interaction network in extramammary Paget’s disease

**DOI:** 10.1186/s12920-021-01135-2

**Published:** 2021-12-11

**Authors:** Da-chao Zheng, Yan-ting Shen, Zi-wei Wei, Xiang Wan, Min-kai Xie, Hai-jun Yao, Zhong Wang

**Affiliations:** Department of Urology, Shanghai 9Th People’s Hospital, Shanghai JiaoTong University School of Medicine, Shanghai, 200011 China

**Keywords:** Extramammary Paget’s disease, RNA-seq, Long non-coding RNA, Expression pattern, Competing endogenous RNAs

## Abstract

**Background:**

Extramammary Paget’s disease (EMPD) is a rare malignant intraepidermal adenocarcinoma that is poorly understood. Regulatory long noncoding RNAs (lncRNAs) are characterized in many species and shown to be involved in processes such as development and pathologies, revealing a new layer of regulation in different diseases, especially in cancer studies. In the present study, we used high-throughput sequencing to reveal the lncRNA–mRNA interaction network in extramammary Paget’s disease.

**Methods:**

High-throughput sequencing was used to identify differentially expressed lncRNA and mRNA profiles between EMPD patients and healthy controls. Then, a series of bioinformatics analyses were conducted to construct the lncRNA–mRNA interaction network, which was finally confirmed in vitro.

**Results:**

Six pairs of EMPD tumor and normal skin samples were collected and sequenced to identify the differentially expressed lncRNA and mRNA profiles between EMPD and healthy controls. A total of 997 differentially expressed mRNAs and 785 differentially expressed lncRNAs were identified. The GO and KEGG analyses show that epidermal development and cell adhesion play important roles in EMPD. The results of the lncRNA–mRNA interaction network analysis suggested that *NEAT1*, *PGAP1*, *FKBP5* and *CDON* were the pivotal nodes of the network and that lncRNA *NEAT1* might regulate mRNA *PGAP1*, *FKBP5* and *CDON*. The results of the quantitative real-time RT–PCR performed in ten other patients for *NEAT1*, *PGAP1*, *FKBP5* and *CDON* were consistent with those of the sequencing analysis. Moreover, an in vitro experiment confirmed the interactions between *NEAT1* and *PGAP1*, *FKBP5* and *CDON* in human immortalized keratinocytes.

**Conclusion:**

These findings suggest that the lncRNA–mRNA interaction network based on four pivotal nodes, *NEAT1*, *PGAP1 FKBP5* and *CDON,* may play an important role in EMPD, which will contribute to a deeper understanding of the pathogenesis of EMPD.

**Supplementary Information:**

The online version contains supplementary material available at 10.1186/s12920-021-01135-2.

## Background

Extramammary Paget’s disease (EMPD) is a rare malignant intraepidermal adenocarcinoma with a high postoperative recurrence rate [1]. It is mostly localized to the vulvar, perianal, scrotal, penile and axillary regions, which are rich in apocrine sweat glands [2, 3]. The initial clinical feature of EMDP is an erythematous plaque of indolent growth with well-defined edges, which is always misdiagnosed as eczema or other chronic dermatitis. Surgical resection, chemotherapy, radiotherapy, photodynamic treatment (PDT) and laser ablation are common treatments [4–6]. However, there are some degrees of limitations in these therapies. For instance, the extremely large areas and multiple separate sites of the lesions make surgical excisions challenging or impractical. Invasive and metastatic EMPD, which does not have curative treatments, always leads to poor outcomes [7]. These limitations require precision medical therapies, such as accurate localization of the lesion area, targeted drugs or tracers, which are impracticable due to the unclear pathogenesis of EMPD. Therefore, exploring the pathogenesis of EMPD is highly significant for both doctors and patients.

Two theories have been proposed: epidermotropic theory and intraepidermal origin theory [8]. The epidermotropic theory postulates that Paget cells originate from underlying adenocarcinoma cells (breast or genitourinary carcinomas) that migrate into the epidermis from skin-associated glands. The intraepidermal origin theory asserts that Paget’s cells are the result of in situ oncogenic changes in epidermal cells of the apocrine gland ducts or pluripotent keratinocyte stem cells rather than migrating adenocarcinoma cells [1]. The intraepidermal origin theory may receive more support as most EMPD lesions are initially limited to the skin and are not accompanied by an underlying adenocarcinoma. However, the true cellular origin has not been revealed thus far. Most outcomes were mainly based on evaluations of patients' clinicopathological characteristics by immunohistochemistry staining, and the pathogenesis has not been revealed [9, 10]. Recently, many researchers have tried to further elucidate the pathogenesis of EMPD by case–control gene and protein expression analysis [11–13]. Several genes, such as *KMT2C*, *FOXA1*, and *GATA3,* were revealed as potential oncogenic genes for Paget’s disease or EMPD. Song and his colleague demonstrated that the Msi1-mTOR signaling pathway might be critical for EMPD pathogenesis through single-cell RNA sequencing [14]. Additionally, microRNAs (miRNAs) could be involved in EMPD development and might be potential serum markers in the diagnosis of EMPD [15, 16]. Currently, regulatory long noncoding RNAs (lncRNAs) have been characterized in many species and are shown to be involved in processes such as development and pathologies, revealing a new layer of regulation in different diseases, especially in cancer studies [17–19]. Thus, integrated analysis of mRNAs, miRNAs, and lncRNAs may reveal novel insights into the exact pathogenesis of EMPD. However, there are few studies about the roles of lncRNA–mRNA interaction networks in the pathogenesis of EMPD, and more research is needed.

Therefore, in this study, to construct the lncRNA–mRNA interaction network associated with EMPD, we first performed high-throughput RNA sequencing for six pairs of EMPD tumor and normal skin tissue samples to identify the differentially expressed (DE) mRNAs and DE lncRNAs, which were subsequently used to construct the lncRNA–mRNA interaction network. Finally, we validated the gene expression level of the pivotal nodes in the lncRNA–mRNA interaction network using quantitative real-time RT–PCR (qRT–PCR) and confirmed their interactions in vitro.

## Results

### Overview of mRNA and lncRNA expression profiles

**Fig. 1:**
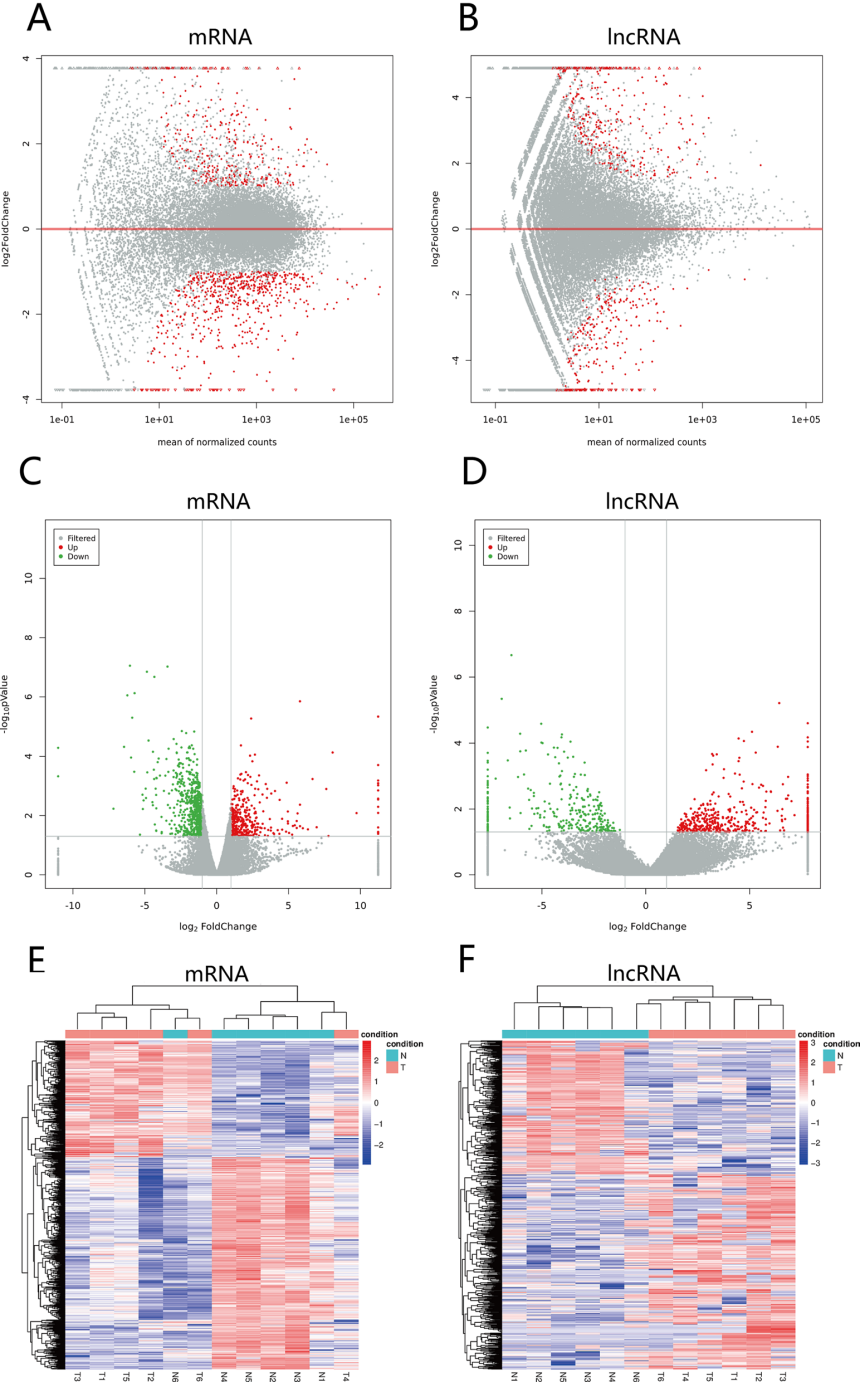
**A**, **B** The differences in mRNAs and lncRNAs produced by the comparison are reflected in the MA plots. The x‐axis is the mean of normalized counts of all samples’ expression, and the y‐axis is the log2 fold-change. The red plots are marked by significantly different genes. **C**, **D** Differential expression analyses of mRNA and lncRNA between cases versus controls. The expression level for each gene was included in the volcano plot. Red and green points indicate the differentially expressed genes (DEGs). Gray and blue points indicated the non‐DEGs. The Y‐axis contains the log10 p value of the genes' mean expression level modified by the DEseq package, and the x‐axis indicates the log2 of the fold changes among two libraries. **E**, **F** Hierarchical clustering results

We analyzed lncRNA and mRNA expression profiles in six pairs of EMPD tumor and normal skin samples using high-throughput RNA-seq analysis. The clinical information of the patients is shown in Table 1. A total of 997 DE mRNAs and 785 DE lncRNAs were identified (p < 0.05, ∣log2FC > 1∣). MA and volcano plots (Fig. 1A–D) were used to assess the expression level of each mRNA and lncRNA between the tumors and controls. The clustering hierarchical results (Fig. 1E,F) revealed that the expression patterns of lncRNAs and mRNAs could largely distinguish EMPD tumors and controls.

**Table 1:** Clinical information of patients

	Case 1	Case 2	Case 3	Case 4	Case 5	Case 6
Gender/age (years)	Male/77	Male/81	Male/74	Male/75	Male/81	Male/71
Delay in diagnosis (month)	24	87	36	120	98	48
Longest diameter of lesion (cm)	9	17	13	7	10	10
Nodule at primary site	No	No	No	Yes	Yes	Yes
Surgical margin status	Negative	Negative	Positive	Negative	Negative	Negative
Lymphovascular invasion	No	No	No	Yes	Yes	No
Regional lymp node metastasis at diagnosis	No	No	–	No	No	No
Dermal invasion	In situ	In situ	In situ	Invasive	Invasive	Invasive

### Functional analyses for DE mRNAs

**Fig. 2:**
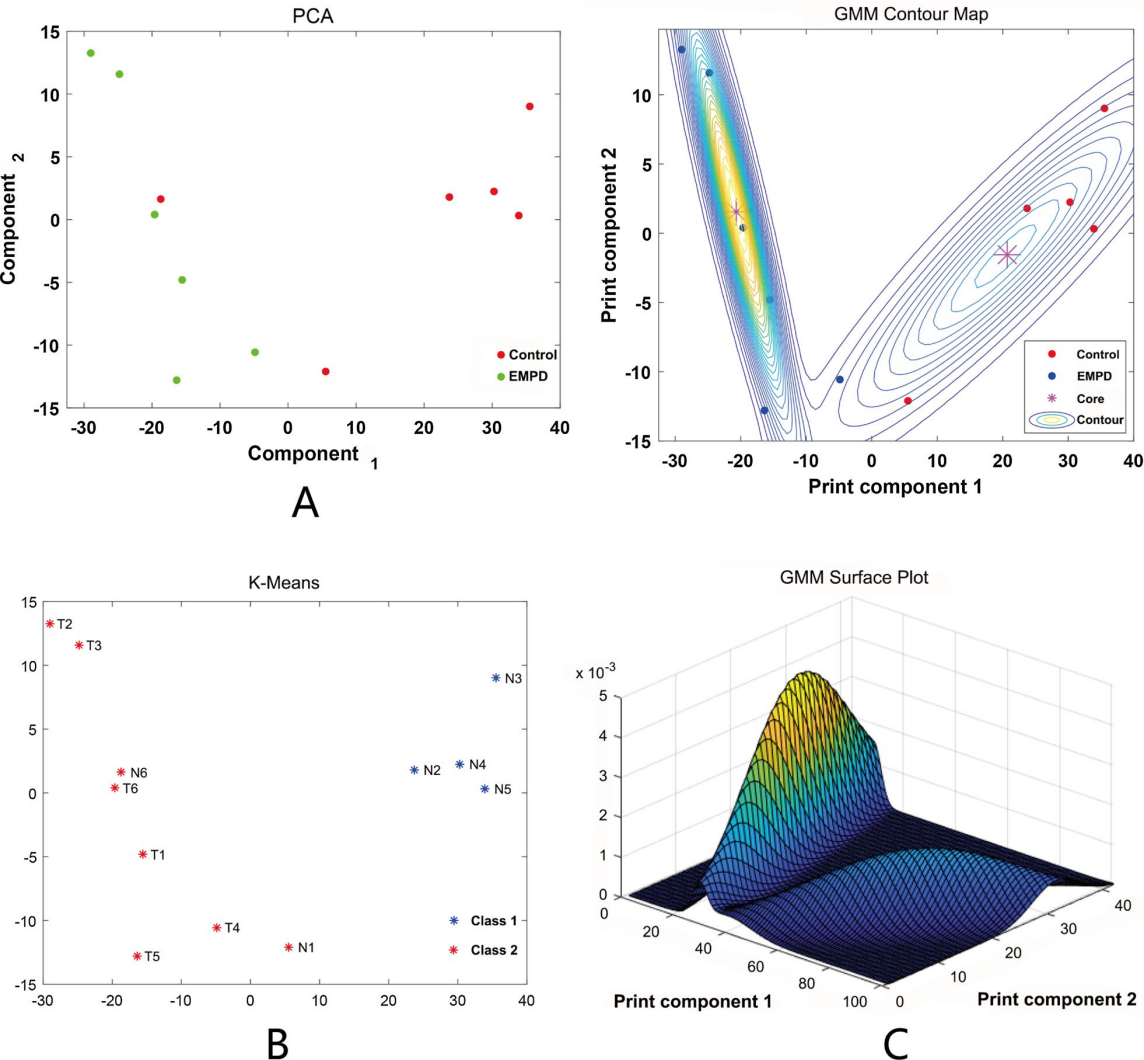
Machine learning for evaluating the ability of DE mRNAs to describe the characteristics of EMPD. **A** PCA; **B** K-Means; **C** GMM

**Fig. 3:**
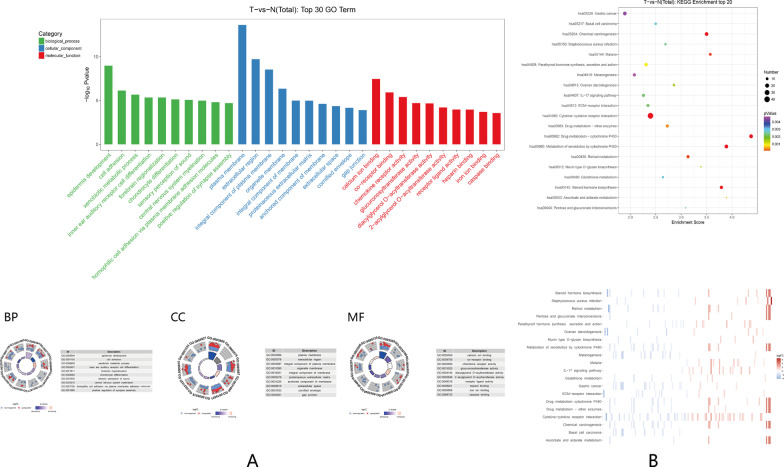
GO and KEGG analyses of the differentially expressed mRNAs. **A** GO annotation of the differentially expressed mRNAs. The bar plot presents the enrichment scores (− log10 [p value]) of the top 10 significantly enriched GO terms in biological processes, cellular components and molecular functions (top); the Z score was calculated to reflect the general expression trend of the DE mRNAs contained in each GO term (bottom). **B** Bulb map of KEGG analysis for the differentially expressed mRNAs (top). The X-axis indicates the enrichment score of differentially expressed genes. Y-axis lists the names of enriched pathways. The size of the node represents the number of enriched differential genes. The P value is represented by a color scale, where the statistical significance increases as purple turns to red. (Bottom) Heatmap for the log2FC of DE mRNAs contained in the significantly enriched KEGG pathways

The functions of lncRNAs are mainly exerted through the regulation of the expression of coding genes [20]. Hence, a series of functional analyses for DE mRNAs were performed. First, machine learning was used to evaluate the ability of DE mRNAs to describe the characteristics of EMPD. The results of PCA and K-means clustering showed that all EMPD samples were clustered in one group (Fig. 2A,B); the GMM results showed that more than 80% (5/6) of EMPD samples had a high probability of being assigned to one group (Fig. 2C). Thus, these DE mRNAs may have a good ability to reflect the features of EMPD. Then, GO and KEGG pathway analyses were performed. A GO analysis was performed for the DE mRNAs to determine which were enriched in GO terms of biological processes (BP), cellular components (CC) and molecular functions (MF). The top 30 GO terms of the three categories are illustrated in Fig. 3A. The GO analysis indicated that several important GO terms were enriched, such as epidermis development, cell adhesion and calcium ion binding. The results of the KEGG pathway enrichment analyses are shown in Fig. 3B. The significantly enriched pathways noted were as follows: (1) cytokine–cytokine receptor interaction; (2) IL-17 signaling pathway; (3) ECM–receptor interaction, and (4) chemical carcinogenesis. Among them, the general expression trend of the DE mRNAs contained in the IL-17 signaling pathway was upregulated, while the general expression trend of the DE mRNAs contained in the ECM–receptor interaction was downregulated.

### Construction of the lncRNA-mRNA interaction network

**Fig. 4:**
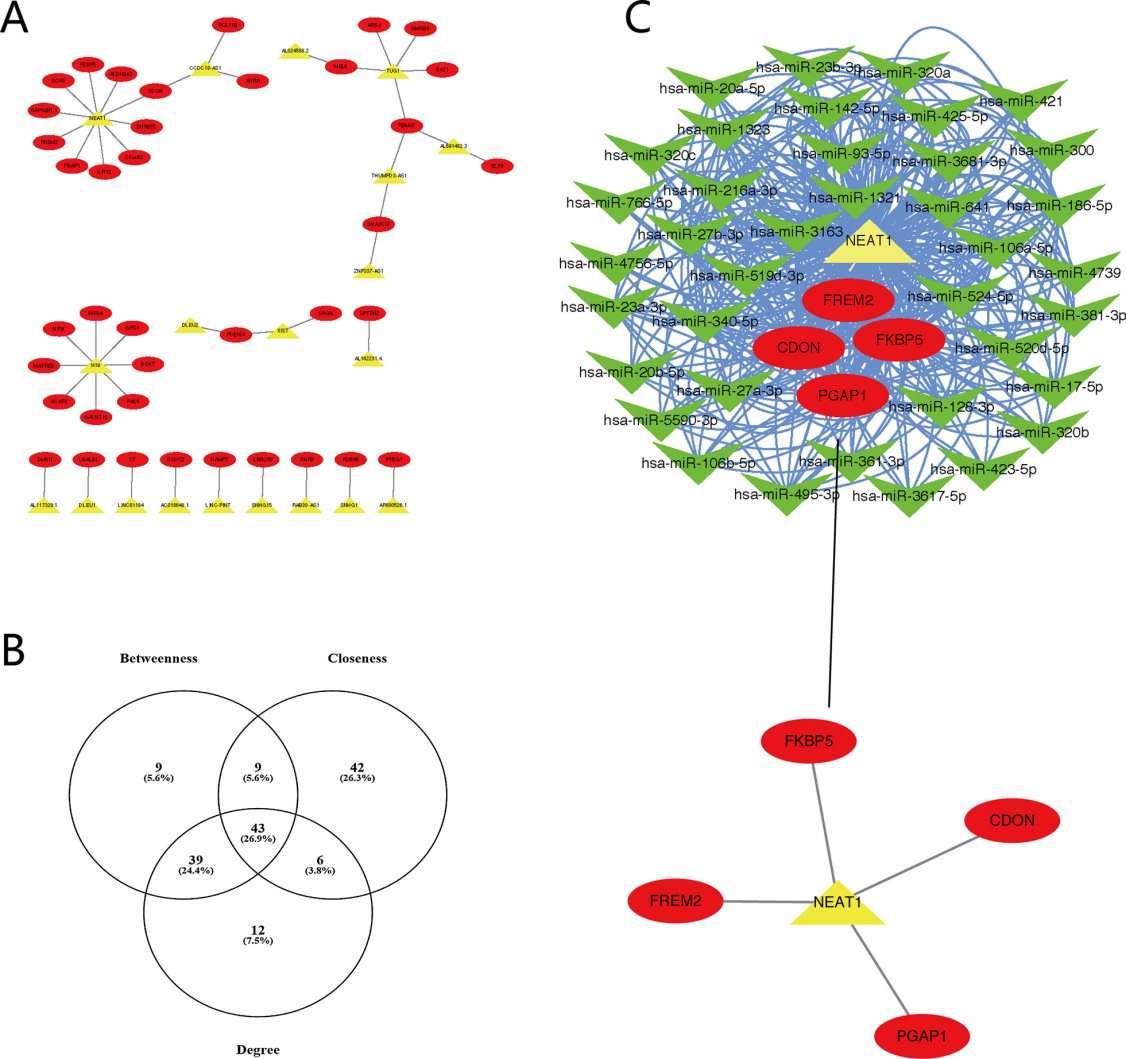
lncRNA–mRNA interaction network of EMPD-related DE genes. **A** DE lncRNAs and mRNAs with significant correlations were merged into a gene–gene interaction network. **B** Forty-three pivotal nodes were listed in this interaction network based on the degree, betweenness centrality and closeness centrality. **C** The interactions of these 43 DE genes, including 1 lncRNA, 38 miRNAs and 4 mRNAs

It is evident that correlations exist between lncRNAs, miRNAs and mRNAs. Pearson’s correlation coefficients (P < 0.05 and r >  = 0.6) and ENCORI were calculated to construct the interaction network between DE lncRNAs and DE mRNAs. A total of 997 DE mRNAs and 786 DE lncRNAs obtained from our sequencing data were used for the lncRNA–mRNA interaction network analysis. Finally, a total of 20 DE lncRNAs and 39 DE mRNAs were included in the network, which was visualized using Cytoscape and is shown in Fig. 4A. Moreover, GO, KEGG, Reactome and WikiPathway enrichment analyses were performed to identify the functions of these 39 mRNAs. The results showed that they probably played roles in nuclear transcription (Additional file 1: Table S2).

To screen the pivotal nodes in the interaction network, a further construction of the mRNA–lncRNA–miRNA interaction network based on the 522 predicted miRNAs was performed. A network tropical analysis was performed, and a Venn diagram was drawn based on the TOP100 degree, TOP100 betweenness centrality and TOP100 closeness centrality of the genes in the network (Fig. 4B). Forty-three genes were found to be the pivotal nodes in the interaction network, and they included one lncRNA (*NEAT1*), four mRNAs (*PGAP1*, *FREM2*, *FKBP5* and *CDON*) and 38 miRNAs (Fig. 4C).

### Validation of sequencing data using qRT–PCR

**Fig. 5:**
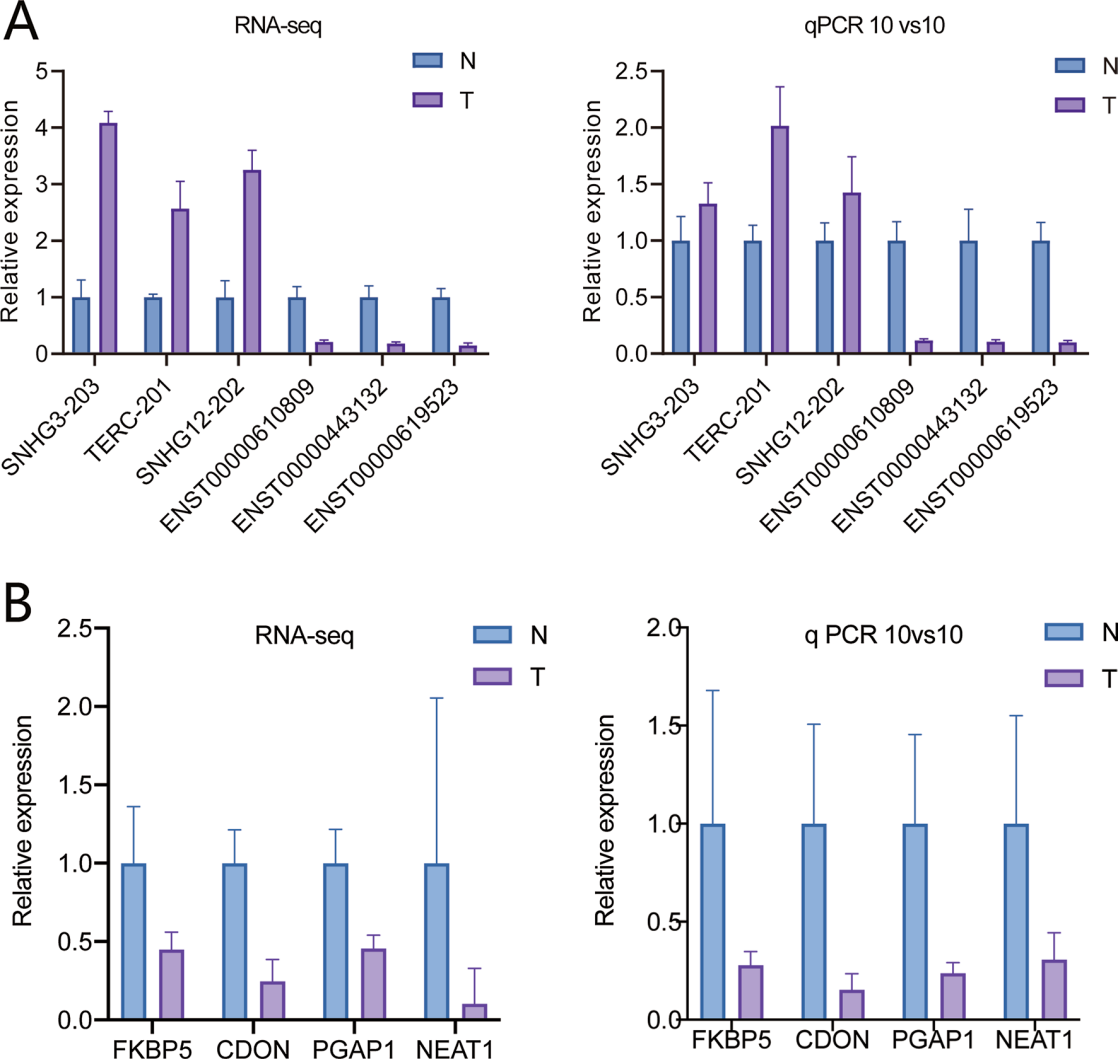
Validation of genes in EMPD tumor and normal skin samples. **A** Validation of 6 random lncRNAs by qRT–PCR. The results showed similar expression patterns in both the validation cohort and sequencing cohort. **B** Another validation of 4 pivotal genes by qRT–PCR. The expression of 4 pivotal genes in the interaction network was determined in a validation cohort. The results showed similar expression patterns in both the validation cohort and sequencing cohort

Six DE lncRNAs were selected randomly to validate the sequencing analysis in the other ten patients using qRT–PCR. The results were consistent with those of the sequencing analyses (Fig. 5A). *SNHG3-203*, *TERC-201* and *SNHG12-202* were upregulated, while *ENST00000610809*, *ENST00000443132* and *ENST00000619523* were downregulated in the EMPD tumor group. Furthermore, the pivotal nodes of the lncRNA–mRNA interaction network, including *NEAT1*, *PGAP1*, *FKBP5* and *CDON*, were also validated by qRT–PCR and found to be downregulated in the EMPD tumor group (Fig. 5B). *FREM2* was not validated because of its extremely low expression in the sequencing analysis. Finally, we used *NEAT1*, *PGAP1*, *FKBP5* and *CDON* to classify the samples. The results showed that all of them had a good ability to identify the EMPD samples from the controls (Additional file 2: Table S2).

### Lentivirus-induced shRNA infection validated the gene–gene interactions

**Fig. 6:**
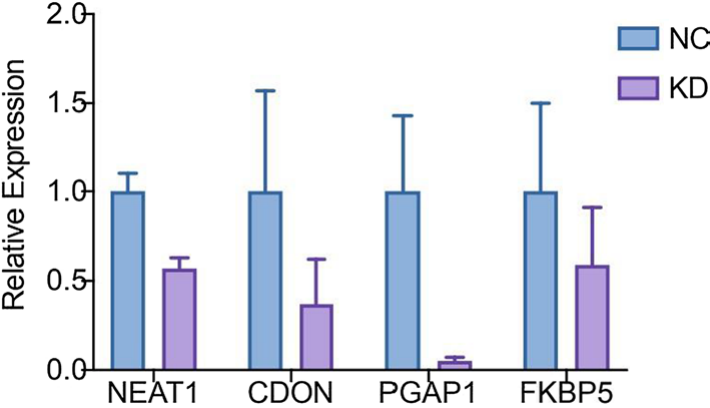
Validation of pivotal gene–gene interactions via shRNA infection. NEAT1 expression level was downregulated after lentivirus-induced shRNA infection, confirming the interaction between these 4 pivotal genes

To validate the gene–gene interactions of the pivotal nodes in the lncRNA–mRNA interaction network, we suppressed the expression of *NEAT1* in human immortalized keratinocytes (HaCaT cells). The qRT–PCR results showed that the expression levels of *PGAP1*, *FKBP5* and *CDON* were also downregulated compared with the vector (Fig. 6), revealing the existing interactions between *NEAT1* and *PGAP1* and between *FKBP5* and *CDON*.

## Discussion

EMPD is an unusual skin neoplasm with unclear pathogenesis. Most studies focused on the expression of markers such as mRNAs, proteins or chemokines but did not reveal their potential pathogenic mechanism [21, 22]. ncRNAs, including ribosomal RNA, transfer RNA, and small nuclear RNA, have been demonstrated to play a crucial role in numerous physiological and pathological processes [23]. LncRNAs have been studied in various diseases for years, and it is widely known that lncRNAs, miRNAs and mRNAs can interact with each other through different mechanisms [24]. However, a comprehensive analysis of lncRNAs and mRNAs in EMPD has not been reported before. Here, we investigated the expression profiles of lncRNAs and mRNAs in EMPD patients using high-throughput sequencing to explore the functions and interactions of lncRNAs and mRNAs. Cellular terms were highly enriched in both the GO and KEGG pathway analyses, including epidermis development and cell adhesion. These results indicated that they probably participated in the pathogenesis of EMPD, such as cancer initiation or invasion.

It is well known that lncRNAs regulate their target mRNA by sponging miRNAs via miRNA response elements (MREs). A competing endogenous RNA (ceRNA) regulatory network forms between lncRNAs, miRNAs and mRNAs [25]. We constructed the lncRNA–mRNA interaction network including 20 lncRNAs, 39 mRNAs and 522 predicted miRNAs. Most of the DE lncRNAs and DE mRNAs have not yet been studied in EMPD, so we analyzed the functions of DE lncRNAs using their targeted mRNAs in the network. A series of functional analyses showed that these DE mRNAs in the lncRNA–mRNA interaction network were mainly involved in nuclear transcription-related processes.

According to the topological analysis of the lncRNA–mRNA interaction network and our validated results, four pivotal nodes, including one lncRNA (*NEAT1*) and three mRNAs (*PGAP1, *FKBP5 and *CDON*), were identified, and they were previously reported to be associated with human tumors, such as non-small-cell lung cancer, prostate cancer, melanoma, and breast cancer [25–29]. In this study, these four pivotal nodes were found to be downregulated, which was in accordance with the results of the GO analysis and the regulatory rules between lncRNAs and mRNAs. The lncRNA *NEAT1* is transcribed from the familial tumoral syndrome multiple endocrine neoplasia type 1 locus, which is located on chromosome 11. It is upregulated in lung cancer, esophageal cancer, breast cancer, and prostate cancer but also downregulated in other diseases, such as acute leukemia and cerebral ischemia [30]. Generally, upregulation of *NEAT1* accounts for the majority. Zhang et al. reported that the upregulation of *NEAT1* activated Wnt/β-catenin signaling and promoted colorectal cancer progression [31]. In contrast, Zhou et al. found that *SIRT1* and *BCL-XL* expression levels were downregulated when miR-377 was upregulated post *NEAT1* knockdown [32]. In addition, Mello et al. reported that *NEAT1* was a p53-regulated large intergenic ncRNA (lincRNA) with a key role in suppressing transformation and cancer initiation [33]. Thus, *NEAT1* deficiency may induce or promote EMPD progression through the p53 signaling pathway.

The three mRNAs, *PGAP1*, *FKBP5* and *CDON*, may act as tumor suppressors in EMPD. CDON is a cell surface receptor associated with cell adhesion and oncogene regulation. CDON expression induces apoptosis but can be inhibited by sonic hedgehog (SHH) [34, 35]. Thus, CDONs can constrain tumor progression by their proapoptotic activity. In existing studies, CDON has been demonstrated to be a negative regulator of several signaling pathways, including Wnt/β-catenin signaling and N-cadherin localization [36–38]. Decreased CDON expression is also observed in a large fraction of human colorectal cancers. As we mentioned, NEAT1 is upregulated in colorectal cancer; thus, the relationship between NEAT1 and CDON in EMPD may be completely different from that in other cancers. PGAP1 (post GPI attachment to protein 1) is a GPI inositol deacylase removing palmitate from inositol. Defects in PGAP1 lead to disorders of psychomotor retardation and facial dysmorphism [39, 40]. Similar to *CDON*, the *PGAP1* protein also regulates WNT signaling [41]. However, this gene has rarely been reported in cancers except gallbladder cholangiocarcinoma [42]. FKBP5 is a glucocorticoid receptor (GR)-binding protein that acts as a cochaperone of heat shock protein 90 (HSP90) and negatively regulates GR [43]. It has been identified to be associated with mental disorders, inflammation and cardiovascular diseases [44, 45]. In cancer etiology and chemoresistance, FKBP5 plays a role in cell apoptosis or death via the glucocorticoid receptor (GR) signaling pathway, NF-kB pathway and AKT–PHLPP pathways [46]. FKBP5 is suggested to be a tumor suppressor in the AKT signaling pathway. This means that downregulation of FKBP5 may also lead to tumors, which has been reported in pancreatic cancer and breast cancer [29, 47].

To our knowledge, this is the first study of a lncRNA–mRNA interaction network for EMPD. In this study, we identified downregulated *NEAT1*, *PGAP1*, *FKBP5* and *CDON* in patient samples, and they were the pivotal nodes in the lncRNA–mRNA interaction network associated with EMPD. In vitro experiments validated the gene–gene interactions between *NEAT1* and *PGAP1*, *FKBP5* and *CDON*, which might play an important role in the pathogenesis of EMPD. Notably, the RNAi experiment was performed with HaCat cells. Due to the lack of a true EMPD cell line, we had to use this homologous, nontumorous epithelial cell line for the validation effort. Although the result may be less rigorous, it could still provide evidence of gene–gene interactions between *NEAT1* and *PGAP1*, *FKBP5* and *CDON* in epithelial cells to a certain degree, including in EMPD. In our future study, an available EMPD cell line is urgently needed.

## Conclusions

We constructed a lncRNA–RNA interaction network consisting of four pivotal nodes, *NEAT1*, *PGAP1*, *FKBP5* and *CDON*, which were significantly downregulated in the tissue samples of EMPD compared with the normal controls. This lncRNA–mRNA interaction network might play crucial roles in the pathogenesis of EMPD.

## Methods

### Sample collection and preparation

EMPD tumor and normal skin samples of six patients diagnosed with EMPD were collected from the specimens during EMPD resections. These six patients were confirmed to have ‘primary’ EMPD using immunostaining for CK7 and CK20. The clinical information of these six patients is shown in Table 1. As validation samples, ten pairs of specimens were collected from patients who were diagnosed with primary EMPD by the Shanghai Ninth Hospital Department of Pathology and stored in liquid nitrogen. The clinical characteristics of these 10 patients are displayed in Additional file 3: Table S3.

### RNA extraction and sequencing analysis

Total RNA was isolated from EMPD tumor and normal skin samples of six patients using TRIzol (Invitrogen, Carlsbad, CA). The RNA concentration and purity were determined using a NanoDrop 2000 spectrophotometer (Thermo Scientific, USA), and later, agarose gel electrophoresis stained with ethidium bromide was used to evaluate the integrity of RNAs. We constructed libraries using the Ribo-Zero Magnetic Gold Kit (Human) (Illumina, San Diego, CA, USA) and NEBNext® Ultra™ RNA Library Prep Kit for Illumina (New England Biolabs) and then sequenced them on the Illumina sequencing platform.

### Bioinformatic analysis

Raw reads were generated by base calling and saved in FASTQ format. Removing reads with adaptors, reads where the number of unknown bases was more than 10% and low-quality reads (the percentage of the low-quality bases with a value ≤ 5 was more than 50% in one read) using FastQC, we generated clean reads for analysis. Then, these clean reads were mapped to the human (GRCh38) genomes through Tophat2 (version 2.0.7) calling Bowtie2 (version 2.1.0) using the default settings. The alignment was performed with Cufflinks (version 2.0.2).

To compare the expression level of a gene across samples, read counts obtained from the RNA‐seq data were normalized as fragments per kilobase of transcript per million mapped fragments (FPKM) [46] with Bowtie 2 [48] and eXpress [20] software packages. FPKM was used to identify differentially expressed genes in the two groups using Cuffdiff. Differences in gene expression (mRNA or lncRNA) with p < 0.05 and |log2FC|> 1 were considered to be significantly differentially expressed.

For functional analyses, enrichment analyses using the GO categories, KEGG pathways [49], and Reactome were utilized to assess and predict the biological functions and signaling pathways of the DE mRNAs and lncRNAs (p < 0.05) using the ‘clusterProfiler’ R package. The results were visualized using the ‘GOplot’ R package.

### Construction of the lncRNA–mRNA interaction network

The interactions between DE mRNAs and lncRNAs were predicted using ENCORI (http://starbase.sysu.edu.cn/). To enhance the reliability, the results were cross-referenced with Pearson’s correlation analysis. Finally, the lncRNA–mRNA interaction network was constructed with Cytoscape software.

### Lentivirus induced shRNA infection in HaCat cells

Human immortalized keratinocytes (HaCat cells) were cultured in DMEM with high glucose supplemented with 10% fetal bovine serum (FBS) at 37 °C. *NEAT1* lentivirus for downregulated expression studies was designed and purchased from Hanbio Biotechnology (Shanghai, China). Lentivirus-packaged shRNA (*NEAT1*), empty vector of lncRNA, and polybrene (5 μg/ml) were cotransfected into HaCaT cells (1.5 × 10^5^ cells per well) according to the manufacturer’s instructions. After 6 h of infection, the supernatant was replaced with new DMEM with 10% FBS. HaCaT cells were cultured for another 48 h and then harvested for quantitative real-time RT–PCR (qRT–PCR) analysis.

### Quantitative real-time PCR

A two-step reaction process, reverse transcription (RT) and PCR, was performed for the quantification of sequencing. A 10-μl mixture including 0.5 μg of RNA, 2 μl of 5 × TransScript All-in-one SuperMix for qPCR and 0.5 μl of gDNA Remover was prepared for each RT reaction. Reactions were performed in a GeneAmp^®^ PCR System 9700 (Applied Biosystems, USA) for 15 min at 42 °C and 5 s at 85 °C. Then, the 10-μl RT reaction mixture was diluted ten times in nuclease-free water and held at − 20 °C. Real-time PCR was performed using a LightCycler^®^ 480 II Real-time PCR Instrument (Roche, Switzerland) with a 10 μl of PCR mixture, which consisted of 1 μl of cDNA, 5 μl of 2 × PerfectStartTM Green qPCR SuperMix (TransGen Biotech, China), 0.2 μl of forward primer, 0.2 μl of reverse primer and 3.6 μl of nuclease-free water. Reactions were incubated in a 384-well optical plate (Roche, Switzerland) at 94 °C for 30 s, followed by 45 cycles of 94 °C for 5 s and 60 °C for 30 s. Each sample was run in triplicate for analysis, and a melting curve analysis was performed for the validation of specific generation of the expected PCR product at the end of the PCR cycles. The primer sequences utilized in this study were designed in the laboratory and are shown in Table 2. The expression levels of mRNAs were normalized to the endogenous control and calculated using the 2^−ΔΔCt^ method.

**Table 2:** The primer sequences used in this study

ID	Primer sequence (5′–3′)	Product length (bp)
FKBP5	F: AGCAGGGAGAGGATATTACC R: TCATCGGCGTTTCCTCAC	87
CDON	F: CTGCATCAGAGACATCAGTCTA R: GGTCCTCATCCGTTTATATTCG	98
PGAP1	F: CCAGATAAATCAGTTGACCCAC R: CTTCAAATGCAGCACTGTAAAG	112
NEAT1	F: TAGCATGTTTGACAGGCGG R: AGTTTAGCGCCAAACCTAGA	126
SNHG3-203	F: TTTGCTTATCAGCTCTTTGTCA R: AGAGGTAAATCCTGACCAACT	86
TERC-201	F: CCTAACTGAGAAGGGCGT R: GCTCTAGAATGAACGGTGG	116
SNHG12-202	F: TGTGACTATGGACCTATGGAG R: GCATGCTGTTGTTTCTACCTAA	89
ENST00000610809	F: CACAAAGGAACGTAGACAAATG R: ATGAGGTGGGCTTGTTTGAA	80
ENST00000443132	F: CTTTGATCTTCTGTGATAGCGA R: TTCTTCCAAGGGGCAGGATAA	107
ENST00000619523	F: ACAGATCATCCTGTCCTCC R: ACACACTCTTCCAAGCAC	86

### Statistical analysis

Statistical analyses were performed with R (version 4.0.5) and SPSS software (v.24.0; IBM, Armonk, NY, USA). Significant differences between the two groups were evaluated by Student’s t test, and correlations between two variables were determined using Pearson’s correlation analysis. In this study, P < 0.05 was regarded as statistically significant. Machine-learning methods, including the Gaussian mixture model (GMM), principal component analysis (PCA) and K-means clustering, were performed using MATLAB® (version 2018b).

## Supplementary Information

**Additional file 1.** The 39 mRNAs in the ceRNA network and their GO, KEGG, Reactome and Wikipathway enrichment analysis.

**Additional file 2.** The results of qRT-PCR analysis showed that NEAT1, PGAP1, FKBP5 and CDON had a well ability to identify the EMPD samples from the controls.

**Additional file 3.** The clinical characteristics of the 10 patients for validation.

## Data Availability

The datasets used and/or analysed during the current study are available from the corresponding author on reasonable request.

## Abbreviations

EMPD: Extramammary Paget’s disease
lncRNA: Long non-coding RNA
PDT: Photodynamic treatment
miRNAs: MicroRNAs
qRT-PCR: Quantitative real-time polymerase chain reaction
DE: Differentially expressed
FPKM: Fragments per kilobase of transcript per million mapped fragments
