# Supplementary material for: Transcriptome sequencing reveals a lncRNA–mRNA interaction network in extramammary Paget’s disease
Source: BMC Med Genomics. 2021 Dec 11;14:291. doi: 10.1186/s12920-021-01135-2 (PMC8665522; doi:10.1186/s12920-021-01135-2)
Supplement: Supplementary file 1 — Additional file 1. The 39 mRNAs in the ceRNA network and their GO, KEGG, Reactome and Wikipathway enrichment analysis. [file 12920_2021_1135_MOESM1_ESM.docx]

| Pathway_Reactome |  |  |  |  |  |  |  |
| --- | --- | --- | --- | --- | --- | --- | --- |
| Gene Set | Description | Size | Expect | Ratio | P Value | FDR | gene |
| R-HSA-162791 | Attachment of GPI anchor to uPAR | 7 | 0.015027 | 66.545 | 0.014935 | 1 | PGAP1 |
| R-HSA-166665 | Terminal pathway of complement | 8 | 0.017174 | 58.227 | 0.017051 | 1 | C7 |
| R-HSA-389599 | Alpha-oxidation of phytanate | 6 | 0.012881 | 77.636 | 0.012815 | 1 | ALDH3A2 |
| R-HSA-392154 | Nitric oxide stimulates guanylate cyclase | 24 | 0.051522 | 38.818 | 0.00118 | 1 | PDE10A, PRKG1 |
| R-HSA-400253 | Circadian Clock | 69 | 0.14813 | 13.502 | 0.0094615 | 1 | CRY2, NAMPT |
| R-HSA-418346 | Platelet homeostasis | 89 | 0.19106 | 10.468 | 0.01539 | 1 | PDE10A, PRKG1 |
| R-HSA-418457 | cGMP effects | 18 | 0.038642 | 51.758 | 0.00065926 | 1 | PDE10A, PRKG1 |
| R-HSA-428157 | Sphingolipid metabolism | 88 | 0.18891 | 10.587 | 0.015064 | 1 | ALDH3A2, ARSJ |
| R-HSA-5632681 | Ligand-receptor interactions | 8 | 0.017174 | 58.227 | 0.017051 | 1 | CDON |
| R-HSA-8937144 | Aryl hydrocarbon receptor signalling | 7 | 0.015027 | 66.545 | 0.014935 | 1 | AHRR |
| [1](http://www.webgestalt.org/results/1607916496/) |  |  |  |  |  |  |  |
|  |  |  |  |  |  |  |  |
| Pathway_KEGG |  |  |  |  |  |  |  |
| Gene Set | Description | Size | Expect | Ratio | P Value | FDR | gene |
| hsa00330 | Arginine and proline metabolism | 50 | 0.12742 | 15.696 | 0.0069902 | 1 | ALDH3A2, SAT1 |
| hsa05032 | Morphine addiction | 90 | 0.22936 | 8.7198 | 0.02152 | 1 | PDE10A, PDE4B |
| hsa00340 | Histidine metabolism | 23 | 0.058615 | 17.06 | 0.057088 | 1 | ALDH3A2 |
| hsa00563 | Glycosylphosphatidylinositol (GPI)-anchor biosynthesis | 23 | 0.058615 | 17.06 | 0.057088 | 1 | PGAP1 |
| hsa00053 | Ascorbate and aldarate metabolism | 27 | 0.068809 | 14.533 | 0.066696 | 1 | ALDH3A2 |
| hsa00512 | Mucin type O-glycan biosynthesis | 27 | 0.068809 | 14.533 | 0.066696 | 1 | GALNT16 |
| hsa00230 | Purine metabolism | 169 | 0.4307 | 4.6437 | 0.067787 | 1 | PDE10A, PDE4B |
| hsa00760 | Nicotinate and nicotinamide metabolism | 30 | 0.076455 | 13.08 | 0.073842 | 1 | NAMPT |
| hsa00410 | beta-Alanine metabolism | 31 | 0.079003 | 12.658 | 0.076212 | 1 | ALDH3A2 |
| hsa04710 | Circadian rhythm | 31 | 0.079003 | 12.658 | 0.076212 | 1 | CRY2 |
|  |  |  |  |  |  |  |  |
|  |  |  |  |  |  |  |  |
| Pathway_Wikipathway |  |  |  |  |  |  |  |
| Gene Set | Description | Size | Expect | Ratio | P Value | FDR | gene |
| WP4222 | Phosphodiesterases in neuronal function | 54 | 0.18079 | 11.062 | 0.013784 | 1 | PDE10A, PDE4B |
| WP2882 | Nuclear Receptors Meta-Pathway | 310 | 1.0379 | 3.854 | 0.018119 | 1 | AHRR, FKBP5, PDE4B, SRGN |
| WP3584 | MECP2 and Associated Rett Syndrome | 71 | 0.23771 | 8.4136 | 0.023131 | 1 | FKBP5, CDON |
| WP4191 | Caloric restriction and aging | 8 | 0.026784 | 37.335 | 0.026487 | 1 | NAMPT |
| WP3630 | NAD metabolism, sirtuins and aging | 10 | 0.03348 | 29.868 | 0.033003 | 1 | NAMPT |
| WP3644 | NAD+ metabolism | 15 | 0.050221 | 19.912 | 0.049112 | 1 | C7 |
| WP4493 | Cells and Molecules involved in local acute inflammatory response | 16 | 0.053569 | 18.668 | 0.052303 | 1 | NAMPT |
| WP4210 | Tryptophan catabolism leading to NAD+ production | 16 | 0.053569 | 18.668 | 0.052303 | 1 | NAMPT |
| WP3413 | NOTCH1 regulation of human endothelial cell calcification | 17 | 0.056917 | 17.57 | 0.055483 | 1 | SAT1 |
| WP2879 | Farnesoid X Receptor Pathway | 19 | 0.063613 | 15.72 | 0.061814 | 1 | FKBP5 |
| [1](http://www.webgestalt.org/results/1607929002/) |  |  |  |  |  |  |  |
| GO_BP |  |  |  |  |  |  |  |
| Gene Set | Description | Size | Expect | Ratio | P Value | FDR | gene |
| GO:0006198 | cAMP catabolic process | 9 | 0.017946 | 111.44 | 0.00013747 | 0.8129 | PDE10A, PDE4B |
| GO:0045944 | positive regulation of transcription by RNA polymerase II | 1128 | 2.2493 | 4.0013 | 0.00025952 | 0.8129 | BCL11B, CDON, ELF3, HIF3A, HMGB3, NAMPT, NFIX, RARB, SOX5 |
| GO:0009214 | cyclic nucleotide catabolic process | 14 | 0.027916 | 71.643 | 0.00034534 | 0.8129 | PDE10A, PDE4B |
| GO:0045893 | positive regulation of transcription, DNA-templated | 1453 | 2.8973 | 3.4515 | 0.00036077 | 0.8129 | BCL11B, CDON, ELF3, HIF3A, HMGB3, NAMPT, NFIX, RARB, SOX5, MAPRE3 |
| GO:1903508 | positive regulation of nucleic acid-templated transcription | 1536 | 3.0628 | 3.265 | 0.00056374 | 0.82029 | BCL11B, CDON, ELF3, HIF3A, HMGB3, NAMPT, NFIX, RARB, SOX5, MAPRE3 |
| GO:1902680 | positive regulation of RNA biosynthetic process | 1537 | 3.0648 | 3.2628 | 0.00056667 | 0.82029 | BCL11B, CDON, ELF3, HIF3A, HMGB3, NAMPT, NFIX, RARB, SOX5, MAPRE3 |
| GO:0021756 | striatum development | 19 | 0.037886 | 52.789 | 0.0006449 | 0.82029 | BCL11B,RARB |
| GO:0030900 | forebrain development | 366 | 0.72981 | 6.8511 | 0.00072809 | 0.82029 | BCL11B, CDON, PGAP1, PRKG1, RARB |
| GO:0051254 | positive regulation of RNA metabolic process | 1620 | 3.2303 | 3.0957 | 0.0008592 | 0.84346 | BCL11B, CDON, ELF3, HIF3A, HMGB3, NAMPT, NFIX, RARB, SOX5, MAPRE3 |
| GO:0048643 | positive regulation of skeletal muscle tissue development | 24 | 0.047856 | 41.792 | 0.0010344 | 0.84346 | CDON, GPC1 |
|  |  |  |  |  |  |  |  |
|  |  |  |  |  |  |  |  |
| GO_MF |  |  |  |  |  |  |  |
| Gene Set | Description | Size | Expect | Ratio | P Value | FDR | gene |
| GO:0030551 | cyclic nucleotide binding | 34 | 0.063484 | 47.256 | 0.000033804 | 0.062268 | PDE10A, PDE4B, PRKG1 |
| GO:0030553 | cGMP binding | 13 | 0.024273 | 82.395 | 0.00025955 | 0.21403 | PDE10A, PRKG1 |
| GO:0004115 | 3',5'-cyclic-AMP phosphodiesterase activity | 15 | 0.028008 | 71.409 | 0.00034858 | 0.21403 | PDE10A, PRKG1 |
| GO:0030552 | cAMP binding | 20 | 0.037344 | 53.557 | 0.00062712 | 0.28879 | PDE10A, PDE4B |
| GO:0004114 | 3',5'-cyclic-nucleotide phosphodiesterase activity | 28 | 0.052281 | 38.255 | 0.0012361 | 0.40712 | PDE10A, PDE4B |
| GO:0004112 | cyclic-nucleotide phosphodiesterase activity | 29 | 0.054148 | 36.936 | 0.0013261 | 0.40712 | PDE10A, PDE4B |
| GO:0043878 | glyceraldehyde-3-phosphate dehydrogenase (NAD+) (non-phosphorylating) activity | 5 | 0.009336 | 107.11 | 0.0093023 | 1 | ALDH3A2 |
| GO:0004028 | 3-chloroallyl aldehyde dehydrogenase activity | 5 | 0.009336 | 107.11 | 0.0093023 | 1 | ALDH3A2 |
| GO:0019808 | polyamine binding | 5 | 0.009336 | 107.11 | 0.0093023 | 1 | SAT1 |
| GO:0003708 | retinoic acid receptor activity | 6 | 0.011203 | 89.261 | 0.011153 | 1 | RARB |

Supplementary table 1: In the ceRNA network, 39 mRNAs were included and GO, KEGG, Reactome and Wikipathway enrichment analyses were performed to identify the function of them. The results showed that they probably played roles in nuclear transcription.
