# Supplementary material for: Transcriptome sequencing reveals a lncRNA–mRNA interaction network in extramammary Paget’s disease
Source: BMC Med Genomics. 2021 Dec 11;14:291. doi: 10.1186/s12920-021-01135-2 (PMC8665522; doi:10.1186/s12920-021-01135-2)
Supplement: Supplementary file 2 — Additional file 2. The results of qRT-PCR analysis showed that NEAT1, PGAP1, FKBP5 and CDON had a well ability to identify the EMPD samples from the controls. [file 12920_2021_1135_MOESM2_ESM.doc]

| No. | Sample | group | (K-means clustering)FLBP5 | (K-means clustering)CDON | (K-means clustering)PGAP1 | (K-means clustering)NEAT1 |
| --- | --- | --- | --- | --- | --- | --- |
| 1 | N1 | control | Class 1 | Class 1 | Class 1 | Class 1 |
| 2 | N10 | control | Class 2 | Class 1 | Class 1 | Class 1 |
| 3 | N2 | control | Class 1 | Class 1 | Class 1 | Class 1 |
| 4 | N3 | control | Class 1 | Class 1 | Class 1 | Class 1 |
| 5 | N4 | control | Class 1 | Class 1 | Class 1 | Class 1 |
| 6 | N5 | control | Class 2 | Class 1 | Class 1 | Class 2 |
| 7 | N6 | control | Class 1 | Class 1 | Class 1 | Class 1 |
| 8 | N7 | control | Class 1 | Class 1 | Class 1 | Class 1 |
| 9 | N8 | control | Class 1 | Class 1 | Class 1 | Class 1 |
| 10 | N9 | control | Class 1 | Class 1 | Class 1 | Class 1 |
| 11 | T1 | EMPD | Class 2 | Class 2 | Class 2 | Class 2 |
| 12 | T10 | EMPD | Class 2 | Class 2 | Class 2 | Class 2 |
| 13 | T2 | EMPD | Class 2 | Class 2 | Class 2 | Class 2 |
| 14 | T3 | EMPD | Class 2 | Class 2 | Class 2 | Class 2 |
| 15 | T4 | EMPD | Class 2 | Class 2 | Class 2 | Class 2 |
| 16 | T5 | EMPD | Class 2 | Class 2 | Class 2 | Class 2 |
| 17 | T6 | EMPD | Class 2 | Class 2 | Class 2 | Class 2 |
| 18 | T7 | EMPD | Class 2 | Class 2 | Class 2 | Class 2 |
| 19 | T8 | EMPD | Class 2 | Class 2 | Class 2 | Class 2 |
| 20 | T9 | EMPD | Class 2 | Class 2 | Class 2 | Class 2 |

Supplementary table 2: The results of qRT-PCR analysis showed that NEAT1，PGAP1，FKBP5 and CDON had a well ability to identify the EMPD samples from the controls
