# Supplementary material for: Transcriptome sequencing reveals a lncRNA–mRNA interaction network in extramammary Paget’s disease
Source: BMC Med Genomics. 2021 Dec 11;14:291. doi: 10.1186/s12920-021-01135-2 (PMC8665522; doi:10.1186/s12920-021-01135-2)
Supplement: Supplementary file 3 — Additional file 3. The clinical characteristics of the 10 patients for validation. [file 12920_2021_1135_MOESM3_ESM.docx]

| Patient | Age（yrs） | Gender | Delay in diagnosis（months） | Longest diameter of lesion（cm） | Nodule at primary site | Surgical margin status | Lymphovascular invasion | Regional lymph node metastasis at diagnosis | Dermal invasion |
| --- | --- | --- | --- | --- | --- | --- | --- | --- | --- |
| Case 1 | 80 | Male | 24 | 8 | Yes | Negative | No | No | Invasive |
| Case 2 | 71 | Male | 120 | 10 | Yes | Negative | Yes | Yes | Invasive |
| Case 3 | 78 | Male | 36 | 11 | No | Negative | No | No | Invasive |
| Case 4 | 72 | Male | 38 | 8 | No | Negative | No | No | In situ |
| Case 5 | 72 | Male | 84 | 14 | Yes | Negative | Yes | Yes | Invasive |
| Case 6 | 67 | Male | 52 | 7 | No | Negative | No | No | In situ |
| Case 7 | 65 | Male | 40 | 8 | Yes | Negative | Yes | Yes | Invasive |
| Case 8 | 66 | Male | 28 | 10 | No | Negative | No | No | In situ |
| Case 9 | 69 | Male | 48 | 5 | No | Negative | No | No | In situ |
| Case 10 | 69 | Male | 108 | 12 | Yes | Negative | No | No | Invasive |

The clinical characteristics of patients for validation
